# 4-[2-(2-Meth­oxy­phen­yl)hydrazinyl­idene]-3-methyl-5-oxo-4,5-dihydro-1*H*-pyrazole-1-carbothio­amide

**DOI:** 10.1107/S1600536811037883

**Published:** 2011-09-30

**Authors:** Hoong-Kun Fun, Suhana Arshad, Shobhitha Shetty, Balakrishna Kalluraya

**Affiliations:** a X-ray Crystallography Unit, School of Physics, Universiti Sains Malaysia, 11800 USM, Penang, Malaysia; b Department of Studies in Chemistry, Mangalore University, Mangalagangothri 574 199, Karnataka, India

## Abstract

In the title mol­ecule, C_12_H_13_N_5_O_2_S, a bifurcated intra­molecular N—H⋯O(O) hydrogen bond forms two *S*(6) ring motifs. The benzene ring forms a dihedral angle of 14.36 (11)° with the pyrazole ring. In the crystal, pairs of N—H⋯S hydrogen bonds form centrosymmetric dimers, generating *R*_2_^2^(8) ring motifs, which stack along the *b* axis.

## Related literature

For applications of pyrazole derivatives, see: Rai *et al.* (2008 ▶); Isloor *et al.* (2009 ▶); Girisha *et al.* (2010 ▶). For standard bond-length data, see: Allen *et al.* (1987 ▶). For hydrogen-bond motifs, see: Bernstein *et al.* (1995 ▶).
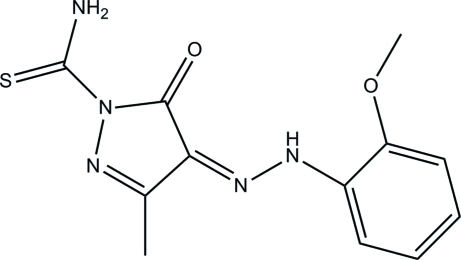


## Experimental

### 

#### Crystal data

C_12_H_13_N_5_O_2_S
*M* *_r_* = 291.33
Monoclinic, 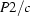

*a* = 14.3207 (13) Å
*b* = 5.2003 (5) Å
*c* = 19.5919 (18) Å
β = 108.369 (2)°
*V* = 1384.7 (2) Å^3^
*Z* = 4
Mo *K*α radiation
μ = 0.24 mm^−1^
*T* = 296 K
0.61 × 0.28 × 0.08 mm

#### Data collection

Bruker SMART APEXII CCD area-detector diffractometer
Absorption correction: multi-scan (*SADABS*; Bruker, 2009 ▶) *T*_min_ = 0.866, *T*_max_ = 0.980
26169 measured reflections
4567 independent reflections
3273 reflections with *I* > 2σ(*I*)
*R*_int_ = 0.061

#### Refinement

*R*[*F* ^2^ > 2σ(*F* ^2^)] = 0.060
*wR*(*F* ^2^) = 0.187
*S* = 1.05
4567 reflections
195 parameters
H atoms treated by a mixture of independent and constrained refinement
Δρ_max_ = 0.60 e Å^−3^
Δρ_min_ = −0.34 e Å^−3^

### 

Data collection: *APEX2* (Bruker, 2009 ▶); cell refinement: *SAINT* (Bruker, 2009 ▶); data reduction: *SAINT*; program(s) used to solve structure: *SHELXTL* (Sheldrick, 2008 ▶); program(s) used to refine structure: *SHELXTL*; molecular graphics: *SHELXTL*; software used to prepare material for publication: *SHELXTL* and *PLATON* (Spek, 2009 ▶).

## Supplementary Material

Crystal structure: contains datablock(s) global, I. DOI: 10.1107/S1600536811037883/lh5336sup1.cif

Structure factors: contains datablock(s) I. DOI: 10.1107/S1600536811037883/lh5336Isup3.hkl

Supplementary material file. DOI: 10.1107/S1600536811037883/lh5336Isup3.cml

Additional supplementary materials:  crystallographic information; 3D view; checkCIF report

## Figures and Tables

**Table 1:** Hydrogen-bond geometry (Å, °)

*D*—H⋯*A*	*D*—H	H⋯*A*	*D*⋯*A*	*D*—H⋯*A*
N1—H1*N*1⋯O2	0.85 (3)	2.13 (3)	2.775 (2)	133 (2)
N5—H2*N*5⋯O2	0.89 (3)	1.98 (3)	2.715 (3)	138 (3)
N5—H1*N*5⋯S1^i^	0.86 (3)	2.52 (3)	3.366 (2)	168 (2)

Symmetry code: (i) 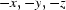.

**Fig. 1.**
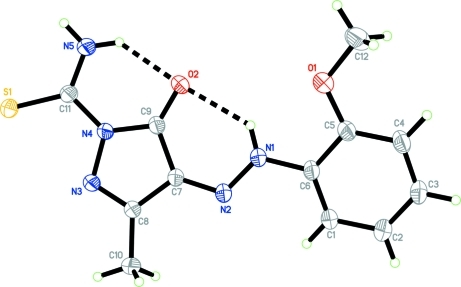
The molecular structure of the title compound, showing 30% probability displacement ellipsoids. The dashed lines indicate intramolecular hydrogen bonds.

**Fig. 2.**
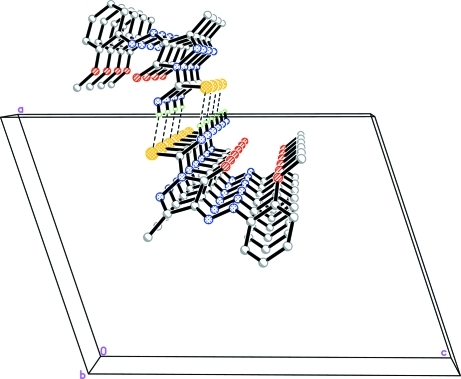
The crystal packing of the title compound. The dashed lines represent the hydrogen bonds.

## supplementary crystallographic information

### Comment

Pyrazoles are a novel class of heterocyclic compounds possessing a wide variety of application in the agrochemical and pharmaceutical industries. Derivatives of pyrazoles are found to show good antibacterial (Rai *et al.*, 2008), anti-inflammatory, analgesic (Isloor *et al.*, 2009), and anticancer activities. Pyrazolines are well known and important nitrogen-containing five membered heterocyclic compounds. Several pyrazoline derivatives have been found to possess considerable biological activities which stimulated research activities in this field (Girisha *et al.*, 2010). In view of these observations and in continuation of our search for biologically active pyrazole derivatives, we herein report the crystal structure of the title compound.

In the molecular structure (Fig. 1), an intramolecular N1—H1N1···O2 and N5—H2N5···O2 hydrogen bond (Table 1) stabilize the molecular structure and forms two *S*(6) ring motifs (Bernstein *et al.*, 1995). The mean planes of the benzene ring (C1–C6) and the 4,5-dihydro-1*H*-pyrazole ring (N3/N4/C7–C9) form a dihedral angle of 14.36 (11)°. Bond lengths (Allen *et al.*, 1987) and angles are within normal range.

The crystal packing is shown in Fig. 2. Molecules are linked by pairs of intermolecular N5—H1N5···S1^i^ hydrogen bonds (Table 1) to form dimers, generating *R*^2^_2_(8) ring motifs (Bernstein *et al.*, 1995) and these sets of ring motifs are stacked along the *b* axis.

### Experimental

To a solution of ethyl 2-[(2-methoxyphenyl)hydrazono]-3-oxobutanoate (0.01 mol) in glacial acetic acid (20 ml), a solution of thiosemicarbazide (0.02 mol) in glacial acetic acid (15 ml) was added and the mixture was refluxed for 4 h. It is cooled and allowed to stand overnight. The solid product that separated out was filtered and dried. It was then recrystallized from ethanol. Crystals suitable for X-ray analysis were obtained from 1:2 mixtures of DMF and ethanol by slow evaporation.

### Refinement

N-bound H atoms was located from the difference map and refined freely, [N–H = 0.85 (3)–0.89 (3) Å]. The remaining H atoms were positioned geometrically [C–H = 0.93 or 0.96 Å] and refined using a riding model with *U*_iso_(H) = 1.2 or 1.5 *U*_eq_(C). A rotating group model was applied to the methyl groups.

### Crystal data

**Table:**

C_12_H_13_N_5_O_2_S	*F*(000) = 608
*M**_r_* = 291.33	*D*_x_ = 1.397 Mg m^−^^3^
Monoclinic, *P*2/*c*	Mo *K*α radiation, λ = 0.71073 Å
Hall symbol: -P 2yc	Cell parameters from 5668 reflections
*a* = 14.3207 (13) Å	θ = 2.2–29.2°
*b* = 5.2003 (5) Å	µ = 0.24 mm^−^^1^
*c* = 19.5919 (18) Å	*T* = 296 K
β = 108.369 (2)°	Plate, red
*V* = 1384.7 (2) Å^3^	0.61 × 0.28 × 0.08 mm
*Z* = 4	

### Data collection

**Table:**

Bruker SMART APEXII CCD area-detector diffractometer	4567 independent reflections
Radiation source: fine-focus sealed tube	3273 reflections with *I* > 2σ(*I*)
graphite	*R*_int_ = 0.061
φ and ω scans	θ_max_ = 31.4°, θ_min_ = 1.5°
Absorption correction: multi-scan (*SADABS*; Bruker, 2009)	*h* = −20→20
*T*_min_ = 0.866, *T*_max_ = 0.980	*k* = −7→7
26169 measured reflections	*l* = −28→28

### Refinement

**Table:**

Refinement on *F*^2^	Primary atom site location: structure-invariant direct methods
Least-squares matrix: full	Secondary atom site location: difference Fourier map
*R*[*F*^2^ > 2σ(*F*^2^)] = 0.060	Hydrogen site location: inferred from neighbouring sites
*wR*(*F*^2^) = 0.187	H atoms treated by a mixture of independent and constrained refinement
*S* = 1.05	*w* = 1/[σ^2^(*F*_o_^2^) + (0.1052*P*)^2^ + 0.245*P*] where *P* = (*F*_o_^2^ + 2*F*_c_^2^)/3
4567 reflections	(Δ/σ)_max_ < 0.001
195 parameters	Δρ_max_ = 0.60 e Å^−^^3^
0 restraints	Δρ_min_ = −0.34 e Å^−^^3^

### Special details

**Table:**

Geometry. All e.s.d.'s (except the e.s.d. in the dihedral angle between two l.s. planes) are estimated using the full covariance matrix. The cell e.s.d.'s are taken into account individually in the estimation of e.s.d.'s in distances, angles and torsion angles; correlations between e.s.d.'s in cell parameters are only used when they are defined by crystal symmetry. An approximate (isotropic) treatment of cell e.s.d.'s is used for estimating e.s.d.'s involving l.s. planes.
Refinement. Refinement of *F*^2^ against ALL reflections. The weighted *R*-factor *wR* and goodness of fit *S* are based on *F*^2^, conventional *R*-factors *R* are based on *F*, with *F* set to zero for negative *F*^2^. The threshold expression of *F*^2^ > σ(*F*^2^) is used only for calculating *R*-factors(gt) *etc*. and is not relevant to the choice of reflections for refinement. *R*-factors based on *F*^2^ are statistically about twice as large as those based on *F*, and *R*- factors based on ALL data will be even larger.

### Fractional atomic coordinates and isotropic or equivalent isotropic displacement parameters (Å^2^)

**Table:**

	*x*	*y*	*z*	*U*_iso_*/*U*_eq_	
S1	0.11741 (4)	0.15498 (9)	0.09216 (3)	0.05009 (17)	
O1	0.17608 (12)	1.2296 (3)	−0.21073 (8)	0.0635 (4)	
O2	0.15180 (10)	0.6859 (3)	−0.08927 (7)	0.0512 (3)	
N1	0.28202 (12)	1.0877 (3)	−0.08210 (8)	0.0464 (4)	
N2	0.32172 (11)	1.0371 (3)	−0.01384 (8)	0.0435 (3)	
N3	0.26013 (11)	0.5730 (3)	0.09587 (7)	0.0431 (3)	
N4	0.19132 (10)	0.5181 (3)	0.02795 (7)	0.0402 (3)	
N5	0.06446 (14)	0.2838 (4)	−0.04505 (9)	0.0567 (5)	
C1	0.40913 (17)	1.3986 (5)	−0.08198 (11)	0.0604 (6)	
H1A	0.4474	1.3460	−0.0364	0.072*	
C2	0.4416 (2)	1.5936 (5)	−0.11722 (13)	0.0735 (7)	
H2A	0.5016	1.6737	−0.0951	0.088*	
C3	0.3850 (2)	1.6686 (5)	−0.18496 (12)	0.0669 (6)	
H3A	0.4073	1.7994	−0.2083	0.080*	
C4	0.29584 (18)	1.5529 (4)	−0.21881 (11)	0.0565 (5)	
H4A	0.2584	1.6049	−0.2647	0.068*	
C5	0.26225 (15)	1.3592 (4)	−0.18418 (9)	0.0469 (4)	
C6	0.31955 (14)	1.2835 (4)	−0.11518 (9)	0.0457 (4)	
C7	0.28270 (13)	0.8477 (3)	0.01271 (9)	0.0405 (4)	
C8	0.31221 (13)	0.7655 (4)	0.08639 (9)	0.0424 (4)	
C9	0.20102 (13)	0.6833 (3)	−0.02533 (9)	0.0399 (3)	
C10	0.38820 (17)	0.8850 (4)	0.14794 (11)	0.0603 (6)	
H10A	0.3937	0.7893	0.1909	0.090*	
H10B	0.3697	1.0591	0.1538	0.090*	
H10C	0.4503	0.8840	0.1389	0.090*	
C11	0.12283 (13)	0.3225 (3)	0.02151 (9)	0.0398 (3)	
C12	0.1117 (2)	1.3083 (6)	−0.27861 (13)	0.0755 (7)	
H12A	0.0572	1.1912	−0.2939	0.113*	
H12B	0.1465	1.3088	−0.3132	0.113*	
H12C	0.0877	1.4782	−0.2747	0.113*	
H1N1	0.231 (2)	1.010 (5)	−0.1081 (15)	0.081 (9)*	
H1N5	0.0188 (18)	0.171 (5)	−0.0500 (14)	0.060 (7)*	
H2N5	0.071 (2)	0.387 (6)	−0.0797 (15)	0.074 (8)*	

### Atomic displacement parameters (Å^2^)

**Table:**

	*U*^11^	*U*^22^	*U*^33^	*U*^12^	*U*^13^	*U*^23^
S1	0.0585 (3)	0.0470 (3)	0.0424 (3)	−0.01645 (19)	0.01250 (19)	0.00532 (18)
O1	0.0680 (9)	0.0748 (10)	0.0413 (7)	−0.0202 (8)	0.0081 (6)	0.0067 (7)
O2	0.0618 (8)	0.0567 (8)	0.0309 (6)	−0.0155 (6)	0.0087 (5)	0.0023 (5)
N1	0.0569 (9)	0.0484 (8)	0.0337 (7)	−0.0151 (7)	0.0141 (6)	0.0030 (6)
N2	0.0532 (8)	0.0441 (8)	0.0353 (7)	−0.0102 (6)	0.0167 (6)	0.0015 (6)
N3	0.0509 (8)	0.0433 (8)	0.0315 (6)	−0.0117 (6)	0.0077 (5)	0.0021 (5)
N4	0.0490 (7)	0.0384 (7)	0.0309 (6)	−0.0117 (6)	0.0095 (5)	0.0003 (5)
N5	0.0659 (10)	0.0568 (10)	0.0396 (8)	−0.0278 (9)	0.0055 (7)	0.0027 (7)
C1	0.0693 (12)	0.0694 (13)	0.0394 (9)	−0.0247 (11)	0.0130 (8)	0.0099 (9)
C2	0.0822 (15)	0.0808 (16)	0.0541 (13)	−0.0374 (13)	0.0167 (11)	0.0121 (11)
C3	0.0923 (16)	0.0626 (13)	0.0486 (11)	−0.0257 (12)	0.0263 (11)	0.0096 (9)
C4	0.0801 (13)	0.0552 (12)	0.0364 (9)	−0.0072 (10)	0.0214 (9)	0.0065 (8)
C5	0.0611 (10)	0.0479 (10)	0.0334 (8)	−0.0078 (8)	0.0173 (7)	−0.0015 (7)
C6	0.0605 (10)	0.0449 (9)	0.0351 (8)	−0.0100 (8)	0.0202 (7)	0.0023 (7)
C7	0.0485 (8)	0.0396 (8)	0.0345 (8)	−0.0097 (7)	0.0148 (6)	0.0002 (6)
C8	0.0498 (9)	0.0432 (9)	0.0330 (7)	−0.0115 (7)	0.0112 (6)	0.0000 (6)
C9	0.0490 (8)	0.0382 (8)	0.0330 (7)	−0.0060 (6)	0.0135 (6)	0.0011 (6)
C10	0.0682 (12)	0.0665 (13)	0.0387 (9)	−0.0277 (10)	0.0060 (8)	0.0003 (9)
C11	0.0457 (8)	0.0332 (8)	0.0394 (8)	−0.0054 (6)	0.0116 (6)	−0.0010 (6)
C12	0.0713 (14)	0.106 (2)	0.0429 (11)	−0.0109 (14)	0.0086 (10)	0.0023 (12)

### Geometric parameters (Å, °)

**Table:**

S1—C11	1.6578 (17)	C1—H1A	0.9300
O1—C5	1.357 (2)	C2—C3	1.375 (3)
O1—C12	1.419 (3)	C2—H2A	0.9300
O2—C9	1.229 (2)	C3—C4	1.378 (3)
N1—N2	1.305 (2)	C3—H3A	0.9300
N1—C6	1.402 (2)	C4—C5	1.383 (3)
N1—H1N1	0.85 (3)	C4—H4A	0.9300
N2—C7	1.317 (2)	C5—C6	1.399 (3)
N3—C8	1.296 (2)	C7—C8	1.435 (2)
N3—N4	1.4131 (18)	C7—C9	1.451 (2)
N4—C11	1.391 (2)	C8—C10	1.482 (2)
N4—C9	1.392 (2)	C10—H10A	0.9600
N5—C11	1.324 (2)	C10—H10B	0.9600
N5—H1N5	0.86 (3)	C10—H10C	0.9600
N5—H2N5	0.89 (3)	C12—H12A	0.9600
C1—C6	1.379 (3)	C12—H12B	0.9600
C1—C2	1.387 (3)	C12—H12C	0.9600
			
C5—O1—C12	117.38 (18)	C1—C6—C5	120.59 (17)
N2—N1—C6	120.92 (16)	C1—C6—N1	122.18 (17)
N2—N1—H1N1	122.0 (19)	C5—C6—N1	117.23 (16)
C6—N1—H1N1	117.0 (19)	N2—C7—C8	126.34 (16)
N1—N2—C7	116.97 (15)	N2—C7—C9	127.67 (16)
C8—N3—N4	106.45 (13)	C8—C7—C9	105.98 (14)
C11—N4—C9	128.13 (14)	N3—C8—C7	111.94 (15)
C11—N4—N3	119.72 (13)	N3—C8—C10	121.01 (16)
C9—N4—N3	112.12 (13)	C7—C8—C10	126.96 (16)
C11—N5—H1N5	115.8 (17)	O2—C9—N4	127.62 (15)
C11—N5—H2N5	118.4 (18)	O2—C9—C7	128.89 (15)
H1N5—N5—H2N5	125 (2)	N4—C9—C7	103.49 (14)
C6—C1—C2	119.4 (2)	C8—C10—H10A	109.5
C6—C1—H1A	120.3	C8—C10—H10B	109.5
C2—C1—H1A	120.3	H10A—C10—H10B	109.5
C3—C2—C1	120.0 (2)	C8—C10—H10C	109.5
C3—C2—H2A	120.0	H10A—C10—H10C	109.5
C1—C2—H2A	120.0	H10B—C10—H10C	109.5
C2—C3—C4	121.02 (19)	N5—C11—N4	114.13 (15)
C2—C3—H3A	119.5	N5—C11—S1	124.15 (14)
C4—C3—H3A	119.5	N4—C11—S1	121.72 (13)
C3—C4—C5	119.66 (19)	O1—C12—H12A	109.5
C3—C4—H4A	120.2	O1—C12—H12B	109.5
C5—C4—H4A	120.2	H12A—C12—H12B	109.5
O1—C5—C4	126.05 (18)	O1—C12—H12C	109.5
O1—C5—C6	114.59 (16)	H12A—C12—H12C	109.5
C4—C5—C6	119.36 (18)	H12B—C12—H12C	109.5
			
C6—N1—N2—C7	−179.10 (17)	N1—N2—C7—C9	1.2 (3)
C8—N3—N4—C11	−178.33 (16)	N4—N3—C8—C7	−0.7 (2)
C8—N3—N4—C9	−0.3 (2)	N4—N3—C8—C10	176.20 (18)
C6—C1—C2—C3	−0.7 (4)	N2—C7—C8—N3	−179.65 (18)
C1—C2—C3—C4	0.1 (4)	C9—C7—C8—N3	1.3 (2)
C2—C3—C4—C5	0.3 (4)	N2—C7—C8—C10	3.7 (3)
C12—O1—C5—C4	−3.6 (3)	C9—C7—C8—C10	−175.4 (2)
C12—O1—C5—C6	176.1 (2)	C11—N4—C9—O2	−1.5 (3)
C3—C4—C5—O1	179.7 (2)	N3—N4—C9—O2	−179.33 (18)
C3—C4—C5—C6	0.0 (3)	C11—N4—C9—C7	178.88 (16)
C2—C1—C6—C5	0.9 (4)	N3—N4—C9—C7	1.00 (19)
C2—C1—C6—N1	−179.2 (2)	N2—C7—C9—O2	0.0 (3)
O1—C5—C6—C1	179.7 (2)	C8—C7—C9—O2	179.03 (19)
C4—C5—C6—C1	−0.6 (3)	N2—C7—C9—N4	179.64 (18)
O1—C5—C6—N1	−0.2 (3)	C8—C7—C9—N4	−1.31 (19)
C4—C5—C6—N1	179.53 (19)	C9—N4—C11—N5	4.3 (3)
N2—N1—C6—C1	12.2 (3)	N3—N4—C11—N5	−177.95 (17)
N2—N1—C6—C5	−167.88 (17)	C9—N4—C11—S1	−176.08 (14)
N1—N2—C7—C8	−177.67 (17)	N3—N4—C11—S1	1.7 (2)

### Hydrogen-bond geometry (Å, °)

**Table:**

*D*—H···*A*	*D*—H	H···*A*	*D*···*A*	*D*—H···*A*
N1—H1N1···O2	0.85 (3)	2.13 (3)	2.775 (2)	133 (2)
N5—H2N5···O2	0.89 (3)	1.98 (3)	2.715 (3)	138 (3)
N5—H1N5···S1^i^	0.86 (3)	2.52 (3)	3.366 (2)	168 (2)

Symmetry codes: (i) −*x*, −*y*, −*z*.
